# Leveraging a nationwide infection surveillance program to implement a colorectal surgical site infection reduction bundle: a pragmatic, prospective, and multicenter cohort study

**DOI:** 10.1097/JS9.0000000000000277

**Published:** 2023-03-14

**Authors:** Josep M. Badia, Nares Arroyo-Garcia, Ana Vázquez, Alexander Almendral, Aina Gomila-Grange, Domenico Fraccalvieri, David Parés, Ana Abad-Torrent, Marta Pascual, Alejandro Solís-Peña, Mireia Puig-Asensio, Miguel Pera, Francesc Gudiol, Enric Limón, Miquel Pujol

**Affiliations:** a Department of Surgery, Hospital General de Granollers, Granollers; b School of Medicine, Universitat Internacional de Catalunya, Sant Cugat del Vallès; c Servei d’Estadística Aplicada, Universitat Autònoma de Barcelona, Bellaterra, Barcelona; d VINCat Program, Catalonia; e Department of Infectious Diseases, Hospital Universitari Parc Taulí, Sabadell; f Department of Surgery, Hospital Universitari de Bellvitge, L’Hospitalet de Llobregat; g Colorectal Surgery Unit, Department of Surgery, Hospital Universitari Germans Trias i Pujol, Badalona, Barcelona; h Universitat Autónoma de Barcelona, Catalonia; i Department of Anaesthesiology, Hospital Universitari Vall d’Hebrón; j Department of Surgery, Hospital del Mar; k Department of Surgery, Hospital Universitari Vall d’Hebrón; l Department of Infectious Diseases, Hospital Universitari de Bellvitge, L’Hospitalet de Llobregat, Barcelona; m Centro de Investigación Biomédica en Red de Enfermedades Infecciosas (CIBERINFEC, CB21/13/00009), Instituto de Salud Carlos III, Madrid; n Department of Surgery, Hospital del Mar; o Universitat de Barcelona; p Department of Infectious Diseases, Hospital Universitari de Bellvitge; q IDIBELL, L’Hospitalet de Llobregat, Barcelona, Spain

**Keywords:** bundle, colorectal surgery, mechanical bowel preparation, normothermia, oral antibiotic prophylaxis, surgical site infection, surveillance program, systemic antibiotic prophylaxis, wound retractor

## Abstract

Bundled interventions usually reduce surgical site infection (SSI) when implemented at single hospitals, but the feasibility of their implementation at the nationwide level and their clinical results are not well established.

**Materials and Methods:**

Pragmatic interventional study to analyze the implementation and outcomes of a colorectal surgery care bundle within a nationwide quality improvement program. The bundle consisted of antibiotic prophylaxis, oral antibiotic prophylaxis (OAP), mechanical bowel preparation, laparoscopy, normothermia, and a wound retractor. Control group (CG) and Intervention group (IG) were compared. Overall SSI, superficial (S-SSI), deep (D-SSI), and organ/space (O/S-SSI) rates were analyzed. Secondary endpoints included microbiology, 30-day mortality, and length of hospital stay.

**Results:**

A total of 37 849 procedures were included, 19 655 in the CG and 18 194 in the IG. In all, 5462 SSIs (14.43%) were detected: 1767 S-SSI (4.67%), 847 D-SSI (2.24%), and 2838 O/S-SSI (7.5%). Overall SSI fell from 18.38% (CG) to 10.17% (IG), odds ratio (OR) of 0.503 [0.473–0.524]. O/S-SSI rates were 9.15% (CG) and 5.72% (IG), OR of 0.602 [0.556–0.652]. The overall SSI rate was 16.71% when no measure was applied and 6.23% when all six were used. Bundle implementation reduced the probability of overall SSI (OR: 0.331; CI_95_: 0.242–0.453), and also O/S-SSI rate (OR: 0.643; CI_95_: 0.416–0.919). In the univariate analysis, all measures except normothermia were associated with a reduction in overall SSI, while only laparoscopy, OAP, and mechanical bowel preparation were related to a decrease in O/S-SSI. Laparoscopy, wound retractor, and OAP decreased overall SSI and O/S-SSI in the multivariate analysis.

**Conclusions:**

In this cohort study, the application of a specific care bundle within a nationwide nosocomial infection surveillance system proved feasible and resulted in a significant reduction in overall and O/S-SSI rates in the elective colon and rectal surgery. The OR for SSI fell between 1.5 and 3 times after the implementation of the bundle.

## Graphical abstract

**Figure:**
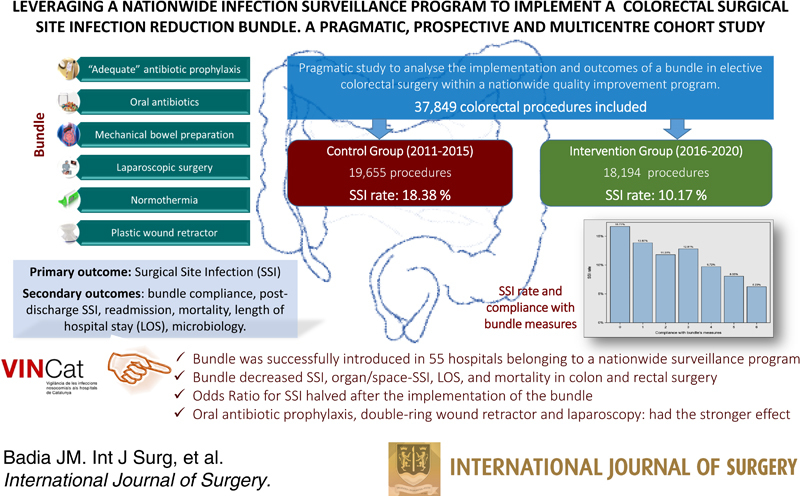

## Introduction

Highlights

- Colorectal surgery has the highest rates of surgical site infection (SSI).
- Bundled interventions usually reduce SSI in colorectal surgery.
- The feasibility of implementing bundles in a large group of hospitals has not been well established, nor has their clinical efficacy.
- A six-measure bundle was successfully introduced in the context of a nationwide healthcare-related infection surveillance system.
- This bundle, which included mechanical and oral antibiotic bowel preparation, lowered rates of SSI in all sites in elective surgery for both the colon and rectum.

Surgical site infections (SSIs) are among the most dreaded postoperative complications and also the most frequent, accounting for 20% of all healthcare-associated infections in Europe^1^. Surgical operations are associated with varying risks of SSI, depending on the underlying clinical diagnosis, the patient’s medical condition, and the type of procedure^2^. Despite the implementation of evidence-based prevention measures, the incidence of SSI after colorectal surgery is the highest among elective abdominal procedures, affecting 15–30% of patients^3^–^9^.

Along with additional surgical procedures, added morbidity, and often higher mortality, SSI places considerable financial strain on the healthcare system owing to the prolonged length of hospital stay (LOS), readmission^10^, and its significant negative impact on patients’ quality of life^11^. In colorectal surgery, organ/space-SSI (O/S-SSI) triples hospital stay and has a readmission rate of 23%, a reoperation rate of 60%, and a 29% rate of need for intensive care^12^.

Although SSIs are a direct consequence of surgery, it is estimated that 60% of them could be prevented with an increased and controlled use of the best evidence-based measures^13^,^14^. Preventive bundles or sets of evidence-based interventions are structured strategies for improving patient outcomes^15^. Some of these intensive quality improvement projects were first implemented for high-risk surgical procedures such as colorectal surgery^16^. However, the adoption of best practice measures within colorectal bundles did not consistently lead to overall SSI reductions^17^–^23^; most have been shown to reduce superficial-SSI, but their impact on deep and O/S-SSI is variable^20^,^24^–^26^. Furthermore, bundles may be easy to introduce in a single hospital, but the feasibility of implementing comprehensive SSI prevention bundles within a larger and more diverse population of hospitals is unclear, and their clinical efficacy has not been well established^27^. Regarding the choice of the components of a colorectal bundle, recent meta-analyses support the efficacy of bundles, including oral antibiotic prophylaxis (OAP), to reduce SSI but also note that certain questions remain unanswered and that well-designed pragmatic studies are needed^28^.

This pragmatic cohort study was designed with the following aims: to assess the feasibility of the implementation of a bundle for SSI reduction in colorectal surgery at the multicenter level in the setting of a nationwide quality improvement program; to evaluate the efficacy of the bundle in reducing SSI in any surgical space; and to examine the association between the degree of bundle adherence and clinical outcomes. Additionally, the study analyses the differences between colon and rectal surgery and the influence of hospital size on SSI outcomes in a large cohort of hospitals.

We hypothesized that a coordinated, guided implementation strategy would allow successful implementation of the bundle and would lower risk-adjusted SSI rates and complications associated with colorectal surgeries at the participating hospitals.

## Material and methods

### Design

This pragmatic, prospective, cohort, multicenter study compares two phases: a baseline period before bundle implementation (Control Group, CG), from January 2011 to June 2016; and the bundle implementation period (Intervention Group, IG), from July 2016 to December 2020.

### Setting and patients

The study uses data collected prospectively within a nationwide infection surveillance system covering a network of public and private hospitals. Data from 55 hospitals participating in the network were included in the analysis. The program is described in detail on the institutional website^29^ and also in previous publications^7^,^30^.

Patients who underwent elective colorectal surgery between January 2011 and December 2020 were included. Cases of elective wound class 2 (clean-contaminated) and 3 (contaminated), according to the National Healthcare Safety Network classification^31^, were followed. Patients with previous ostomies or peritonitis at the time of intervention (wound class 4) were excluded. Table 1 shows in detail the inclusion criteria for colorectal surgery surveillance. Prospective surveillance was performed by training the infection control team (ICT) at each hospital to ensure appropriate data collection. A detailed operational definition document was generated and shared with all network hospitals. The definitions, criteria, and surveillance methodology used by the ICT staff were identical in the two study periods. The ICTs received prior training to ensure consistent and accurate data collection, and audits of the data provided were conducted at different times during the development of the program. Active mandatory postdischarge surveillance was performed up to day 30 postsurgery.

**Table 1:** Inclusion and exclusion criteria for colorectal surgery surveillance.

Inclusion criteria Colon or rectal elective resection surgery (all diseases that require surgical resection are included: malignant and benign neoplastic diseases, chronic inflammatory disease, and diverticulosis). Delayed surgery (patient admitted as an emergency, but surgery performed on a scheduled basis during the same hospital admission, for example colonic bowel obstruction treated with an endoscopic stent and operated days later) Elective wound class 2 (Clean-contaminated) and 3 (Contaminated) cases. Minimum of 100 consecutive procedures per year per hospital or continuous monitoring throughout the year for those centers that perform fewer than 100 procedures per year.
Exclusion criteria Emergency surgery. Peritonitis at the time of intervention (wound class 4 surgery). Patients who underwent multiple procedures during the surgery itself, for example resection of liver metastases (until 2015). From 2016, cases with other procedures that can accompany colon surgery, such as cholecystectomy, herniorrhaphy, appendicectomy, nephrectomy, liver segmentectomy, or partial bladder resection were included. Patients with previous ostomies. Centers that performed fewer than 10 surgical procedures annually.

### Intervention

A multidisciplinary team of nurses and medical and surgical specialists was recruited to formulate a bundle of preventive measures specific to colorectal surgery. The literature for optimal care during the preoperative, intraoperative, and postoperative phases was reviewed, including evidence on OAP and mechanical bowel preparation (MBP)^32^. Practices were chosen either for their high level of scientific evidence or for being considered reasonable, associated with minimal risk, and potentially beneficial. On this basis, the working group created a 6-measure bundle to be implemented voluntarily by the participating hospitals. The measures in the bundle were adequate antibiotic intravenous prophylaxis (antibiotic type, dose, timing within 60 min, intraoperative re-dosing, and duration <24 h), OAP, MBP, laparoscopic surgery, maintenance of normothermia (goal >36°C), and the use of a double-ring plastic wound retractor in open and laparoscopic surgery (Table 2).

**Table 2:** Measures included in the colorectal bundle.

‘Adequate’ systemic iv antibiotic prophylaxis	‘Adequate’=all the following items must be fulfilled. Start 30–60 min before incision. Intraoperative re-dosing when indicated. Do not prolong >24 h. Type of antibiotic according to hospital protocol. *Recommended*: • Metronidazole 15 mg/kg+gentamycin 5 mg/kg or • Cefuroxime 1.5 g+metronidazole 15 mg/kg or • Cefazolin 2 g+metronidazole 15 mg/kg or • Amoxicillin-clavulanate 2 g
Mechanical bowel preparation	Day before the procedure
Oral antibiotic prophylaxis	Day before the procedure. *Recommended*: • Metronidazole 750 mg+neomycin 1 g (three doses the day before surgery). or • Erythromycin 1 g+metronidazole 750 mg (three doses the day before surgery)
Laparoscopic surgery	
Maintenance of normothermia	Goal: >36° at the end of operation
Double-ring plastic wound edge retractor	In open or laparoscopic surgery

The intervention began on 1 January 2016, with the dissemination of the bundle measures via e-mail to all participating hospitals, and a workshop addressed to the surgical and ICTs. Hospitals were given the option to implement either all or a set of individual bundle components. The bundle involved a systematic approach to improving the use of SSI preventive measures across the phases of perioperative care. It was a multidisciplinary project in which surgeons, anaesthesiologists, surgical nurses, operating room staff, unit nurses, house staff, and hospital mid-level providers were asked to enact the prescribed elements. Participating institutions created local improvement teams with the support of senior leaders from the hospital to facilitate the implementation of SSI prevention measures.

### Study outcomes, variables, definitions, and data source

Basic demographic data were recorded, including age, gender, American Society of Anaesthesiologists (ASA) score, and information on surgical details, including surgical approach, wound contamination class, and duration of surgery. The National Nosocomial Infections Surveillance (NNIS) score was also calculated for each patient.

The primary outcome was the development of a SSI within 30 days after operation, according to the Centers for Disease Control and Prevention (CDC) definitions^33^. SSIs were defined as superficial incisional (S-SSI), deep incisional (D-SSI), and organ space (O/S-SSI). The term ‘overall SSI’ refers to the sum of the SSI at all three anatomical levels. When necessary, ‘incisional SSI’ (I-SSI) means the addition of S-SSI and D-SSI. The incidence of SSI was measured as events per 100 procedures included.

Secondary outcome variables included postdischarge SSI, readmission, postoperative 30-day mortality, LOS, time from surgical procedure to SSI, microbiological etiology of infections, and compliance with the bundle of six perioperative measures.

### Ethical issues

The implementation of the bundle precluded randomization. The data were taken from a large nonpublicly available national database. Patients’ confidential information was protected in accordance with European regulations. Anonymity and data confidentiality (access to records, data encryption, and archiving of information) were maintained throughout the research process. Data extraction was approved by the Institutional Research Board, and the study was approved by the Clinical Research Ethics Committee. The need for informed consent and the provision of an information sheet were waived because data were routinely collected as part of hospital surveillance and quality improvement. The project has the Research Registry UIN: researchregistry8407 at https://www.researchregistry.com (https://www.researchregistry.com/browse-the-registry#home/registrationdetails/634d398305178e002191c978/) and was also registered with ClinicalTrials.gov Identifier: NCT04129177 (https://clinicaltrials.gov/ct2/show/NCT04129177). The study has been reported in accordance with the STROCSS (Strengthening the reporting of cohort, cross-sectional and case-control studies in surgery) criteria^34^.

### Statistical analysis

Descriptive statistical analyses were performed using frequencies and proportions for categorical variables, while medians and interquartile range (IQR) or means and SD were used for continuous variables. Infection rates were expressed as cumulative incidence, that is, the crude percentage of operations resulting in SSI/number of surgery procedures. Furthermore, some analyses were stratified by year, risk index category, hospital size, and SSI type. Spearman correlation coefficient (*ρ*) was used to describe the evolution of infection rates and mortality over the years. Any relationship between two qualitative variables was analyzed using contingency tables and performing the χ^2^ test or the likelihood ratio test as appropriate.

A univariate logistic regression model was performed to analyze the individual effects of the bundle measures, and a multinomial logistic regression model was performed to study the combined effect of all bundle measures over the years.

The results are presented in terms of OR (estimated infection rates), with the corresponding 95% confidence intervals (CI_95_). The significance level was set at 5% in all tests. The results are analyzed using the statistical package SAS v9.4 (SAS Institute Inc., Cary, North Carolina, USA).

## Results

The study included 37 849 patients, 19 655 in the CG (13 886 colon surgery and 5769 rectal surgery) and 18 194 in the IG (13 363 colon surgery and 4831 rectal surgery). The demographic and baseline characteristics of the two cohorts are displayed in Table 3.

**Table 3:** Characteristics of patients in Control Group and Inter**v**ention Group.

	Overall	Control group	Intervention group	*P*
Colorectal surgery				
Number of procedures	37 849	19 655	18 194	
Wound class				<0.0001
3 (clean/contaminated)	36 883 (97.60%)	18 930 (96.40%)	17 953 (89.90%)	
4 (contaminated)	906 (2.40%)	706 (3.60%)	200 (1.10%)	
Age, years (mean, SD)	68.67 (12.45)	68.73 (12.41)	68.61 (12.50)	0.3231
Sex, male (%)	22 690 (59.95%)	11 899 (60.54%)	10 791 (59.31%)	0.0148
Median duration of intervention, minutes (Q1, Q3)	165 (125, 220)	165 (120, 216)	170 (129, 225)	<0.0001
ASA score				0.0007
ASA score 1	1979 (5.27%)	1098 (5.59%)	881 (4.91%)	
ASA score 2	20 827 (55.44%)	10 826 (55.16%)	10 001 (55.74%)	
ASA score 3	13 895 (36.99%)	7207 (36.72%)	6688 (37.28%)	
ASA score 4	858 (2.28%)	492 (2.51%)	366 (2.04%)	
Laparoscopy (%)	25 069 (66.51%)	11 493 (58.68%)	13 576 (74.97%)	<0.0001
NNISS ≥1 (%)	11 507 (30.40%)	6646 (33.81%)	4861 (26.72%)	<0.0001
Colon surgery				
Number of procedures	27 249	13 886	13 363	
Wound class				<0.0001
3 (clean/contaminated)	26 671 (98.02%)	13 434 (96.86%)	13 237 (99.23%)	
4 (contaminated)	538 (1.98%)	435 (3.14%)	103 (0.77%)	
Age, years (mean, SD)	69.10 (12.41)	69.09 (12.38)	69.10 (12.44)	0.9851
Sex, male (%)	15 845 (58.15%)	8174 (58.87%)	7671 (57.40%)	0.0146
Median duration of intervention, minutes (Q1, Q3)	154 (120, 200)	150 (115, 195)	157(120, 204)	<0.0001
ASA score				0.0027
ASA score 1	1430 (5.29%)	774 (5.58%)	656 (4.97%)	
ASA score 2	14 899 (55.07%)	7547 (54.44%)	7353 (55.73%)	
ASA score 3	10 056 (37.17%)	5158 (37.21%)	4898 (37.13%)	
ASA score 4	665 (2.46%)	380 (2.74%)	284 (2.16%)	
Laparoscopy (%)	18 082 (66.63%)	8103 (58.57%)	9979 (75.01%)	<0.0001
NNISS ≥1 (%)	8311 (30.50%)	4756 (34.25%)	3555 (26.60%)	<0.0001
Rectal surgery				
Number of procedures	10 600	5769	4831	
Wound class				<0.0001
3 (clean/contaminated)	10 212 (96.52%)	5496 (95.30%)	4716 (97.98%)	
4 (contaminated)	368 (3.48%)	262 (4.70%)	97 (2.02%)	
Age, years (mean, SD)	67.59 (12.49)	67.87 (12.43)	67.26 (12.56)	0.0120
Sex, male (%)	6845 (64.58%)	3725 (64.57%)	3120 (64.58%)	0.9883
Median duration of intervention, minutes (Q1, Q3)	210 (155, 270)	205 (150, 265)	215 (160, 276)	<0.0001
ASA score				0.0161
ASA score 1	549 (5.22%)	324 (5.62%)	225 (4.97%)	
ASA score 2	5928 (56.39%)	3279 (56.89%)	2649 (55.79%)	
ASA score 3	3839 (36.52%)	2049 (35.55%)	1790 (37.70%)	
ASA score 4	193 (1.84%)	112 (1.94%)	81 (1.71%)	
Laparoscopy (%)	6987 (66.19%)	3390 (58.95%)	3597 (74.86%)	<0.0001
NNISS ≥1 (%)	3196 (30.15%)	1890 (32.76%)	1306 (27.03%)	<0.0001

Adequate surgical prophylaxis: type of antibiotic according to local guidelines, in addition to correct timing, dosage, and duration.

ASA, American Society of Anesthesiologists; NNISS, National Nosocomial Infections Surveillance System Index; Q1, first quartile; Q3, third quartile.

### SSI rates

#### Overall colorectal surgery

**Figure 1:**
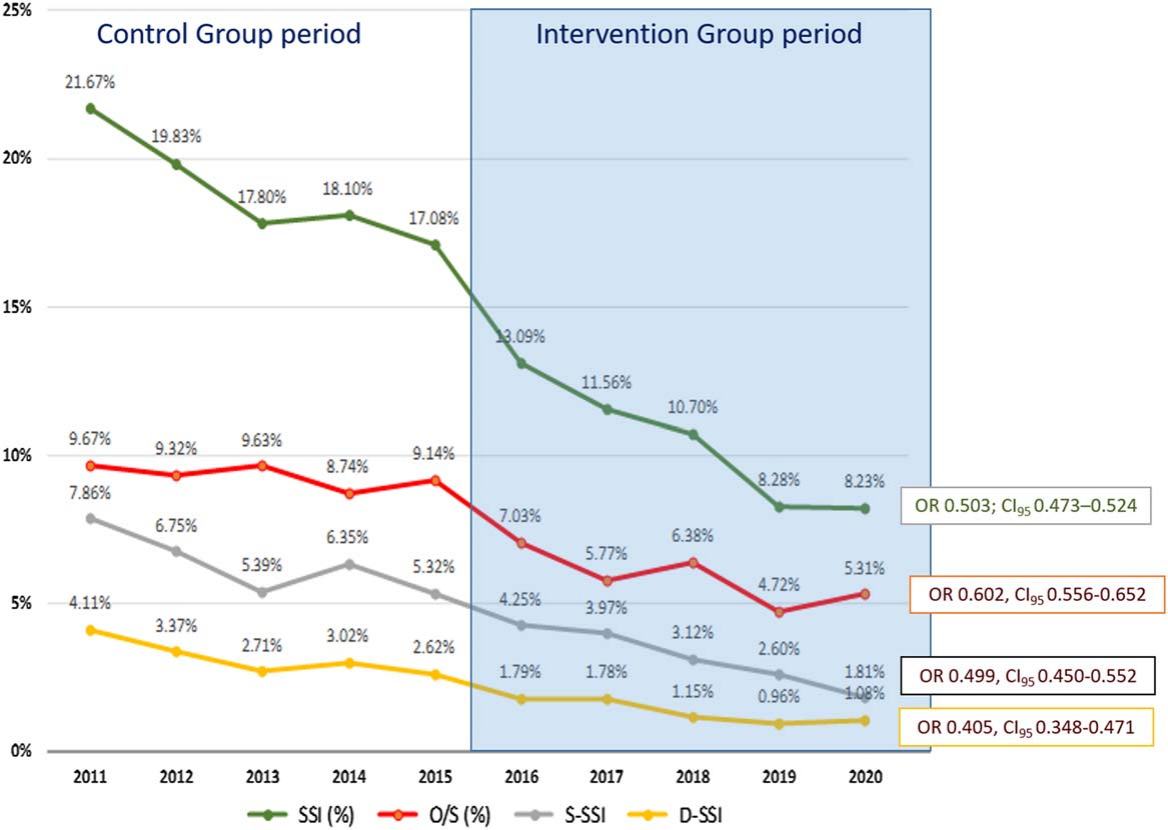
Aggregate colorectal SSI, and superficial, deep, and organ/space-SSI rates during the period of the study (2011–2020). SSI, surgical site infection.

Figure 1 shows the trends of SSI incidence over the course of the study period. There were 5462 SSIs, representing a cumulative incidence of 14.43%. This incidence fell significantly over the years (*ρ*=−0.98788). With regard to the surgical site affected, 1767 (4.67%) infections were S-SSI, 847 (2.24%) D-SSI, and 2838 (7.50%) O/S-SSI (Table 4 and Fig. 1).

**Table 4:** Overall SSI rates and comparison of SSI rates in the pragmatic trial.

	Overall (%)	Control group (%)	Intervention group (%)	OR [CI_95%_]	*P*
Colorectal surgery	14.43	18.38	10.17	0.503 [0.473–0.534]	<0.0001
Superficial-SSI	4.67	6.09	3.13	0.499 [0.450–0.552]	<0.0001
Deep-SSI	2.24	3.12	1.29	0.405 [0.348–0.471]	<0.0001
O/S-SSI	7.50	9.15	5.72	0.602 [0.556–0.652]	<0.0001
Colon surgery	13.05	17.09	8.85	0.471 [0.437–0.507]	<0.0001
Superficial-SSI	4.64	6.30	2.92	0.447 [0.396–0.505]	<0.0001
Deep-SSI	1.73	2.44	0.99	0.399 [0.326–0.488]	<0.0001
O/S-SSI	6.65%	8.32	4.90	0.568 [0.514–0.627]	<0.0001
Rectal surgery	17.99	21.48	13.83	0.587 [0.529–0.650]	<0.0001
Superficial-SSI	4.74	5.58	3.73	0.655 [0.543–0.789]	<0.0001
Deep-SSI	3.55	4.75	2.11	0.433 [0.343–0.545]	<0.0001
O/S-SSI	9.69	11.13	7.97	0.692 [0.606–0.790]	<0.0001

O/S-SSI, organ space-surgical site infection; SSI, surgical site infection.

**Figure 2:**
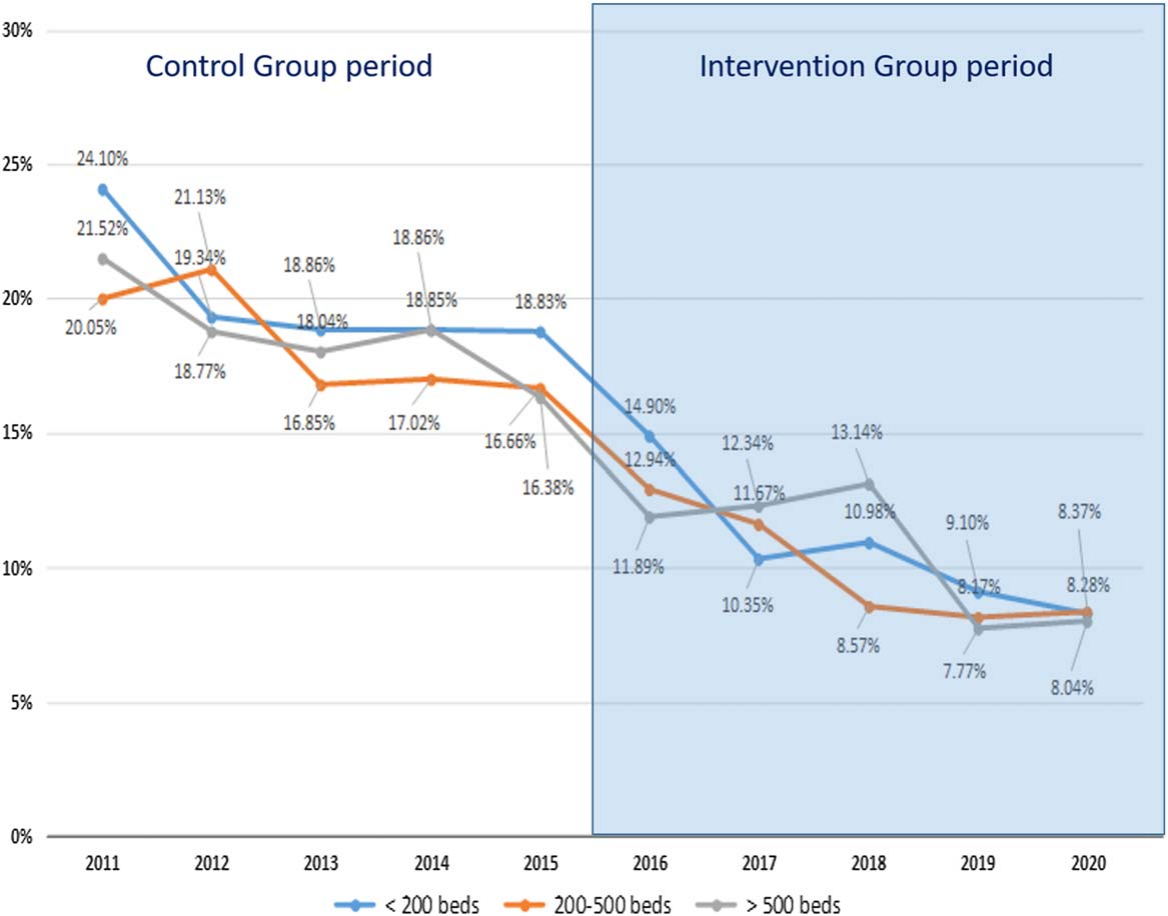
Aggregate colorectal surgical site infection rates according to hospital size.

Comparing the two study groups, the overall SSI rate for colorectal surgery was 18.38% in the CG and 10.17% in the IG (OR: 0.503 [CI_95_: 0.473–0.534]; *P*<0.0001). In all locations, SSI fell significantly, in O/S-SSI it was 9.15% in the CG and 5.72% in the IG (OR: 0.602; CI_95_: 0.556–0.652; *P*<0.0001). The decrease in overall and O/S-SSI rates was similar in high-volume, medium-volume, and low-volume hospitals, as shown in Figure 2.

#### Colon surgery

The overall SSI rate was 17.09% in the CG and 8.85% in the IG (OR: 0.471 [CI_95_: 0.437–0.507]; *P*<0.0001). The trend in SSI rates was also significant, with *ρ* −0.97576 (Table 4). O/S-SSI rates were 8.32% in the CG and 4.90% in the IG (OR: 0.568 [CI_95_: 0.514–0.627]); *P*<0.0001), with Spearman coefficient −0.95152.

#### Rectal surgery

The overall SSI rate was 21.48% in the CG and 13.83% in the IG, OR of 0.587 [CI_95_: 0.529–0.650]; *P* less than 0.0001. The overall SSI decrease in rectal surgery was also significant, with *ρ* −0.96364 (Table 4). O/S-SSI rates were 11.13% in the CG and 7.97% in the IG (OR: 0.692 [CI_95_: 0.606–0.790]; *P*<0.0001), with a significant, but less evident decrease (*ρ* –0.72121).

### Bundle adherence and SSI rates

Table 5 shows the percentage of use of the measures. The rates of correct compliance with each measure were 82.37% for IV prophylaxis, 74.97% for laparoscopy, 92.23% for maintenance of normothermia, 73.78% for OAP, 78.87% for MBP, and 75.61% for wound protection. The level of adherence to each recommendation of the bundle did not differ according to the type of surgery. Comparing the two periods of the study, the use of laparoscopy increased in both colon (58.57% vs. 75.01%; *P*<0.0001) and rectal surgery (58.95% vs. 74.86%; *P*<0.0001).

**Table 5:** Percentage of use of bundle measures in the study groups.

	Control group, *N* (%)	Intervention group, *N* (%)	*P*
Colorectal surgery			
Adequate antibiotic prophylaxis*	16 701 (86.91)	14 965 (82.37)	<0.001
Oral antibiotic prophylaxis (OAP)	NA	8868 (73.78)	
Mechanical bowel preparation (MBP)	NA	9744 (78.87)	
Laparoscopy	11 493 (58.68)	13 576 (74.97)	<0.001
Maintenance of normothermia	NA	10 135 (92.23%)	
Double-ring wound retractor	NA	8781 (75.61%)	
Colon surgery			
Adequate antibiotic prophylaxis*	11 850 (87.25%)	11 048 (82.76%)	<0.001
OAP	NA	6326 (71.22%)	
MBP	NA	6761 (73.89%)	
Laparoscopy	8103 (58.57%)	9979 (75.01%)	<0.001
Maintenance of normothermia	NA	7558 (92.32%)	
Double-ring wound retractor	NA	6876 (78.97%)	
Rectal surgery			
Adequate antibiotic prophylaxis*	4851 (86.09%)	3917 (81.28%)	<0.001
OAP	NA	2542 (81.03%)	
MBP	NA	2983 (93.10)	
Laparoscopy	3390 (58.95%)	3597 (74.86%)	<0.001
Maintenance of normothermia	NA	2577 (91.97%)	
Double-ring wound retractor	NA	1895 (65.50%)	

Only information on the adequation of systemic antibiotic prophylaxis and the use of laparoscopy was available in the period before the implementation of the bundle.

* Adequate surgical prophylaxis: type of antibiotic according to local guidelines, in addition to correct timing, dosage and duration.

NA, not available.

**Figure 3:**
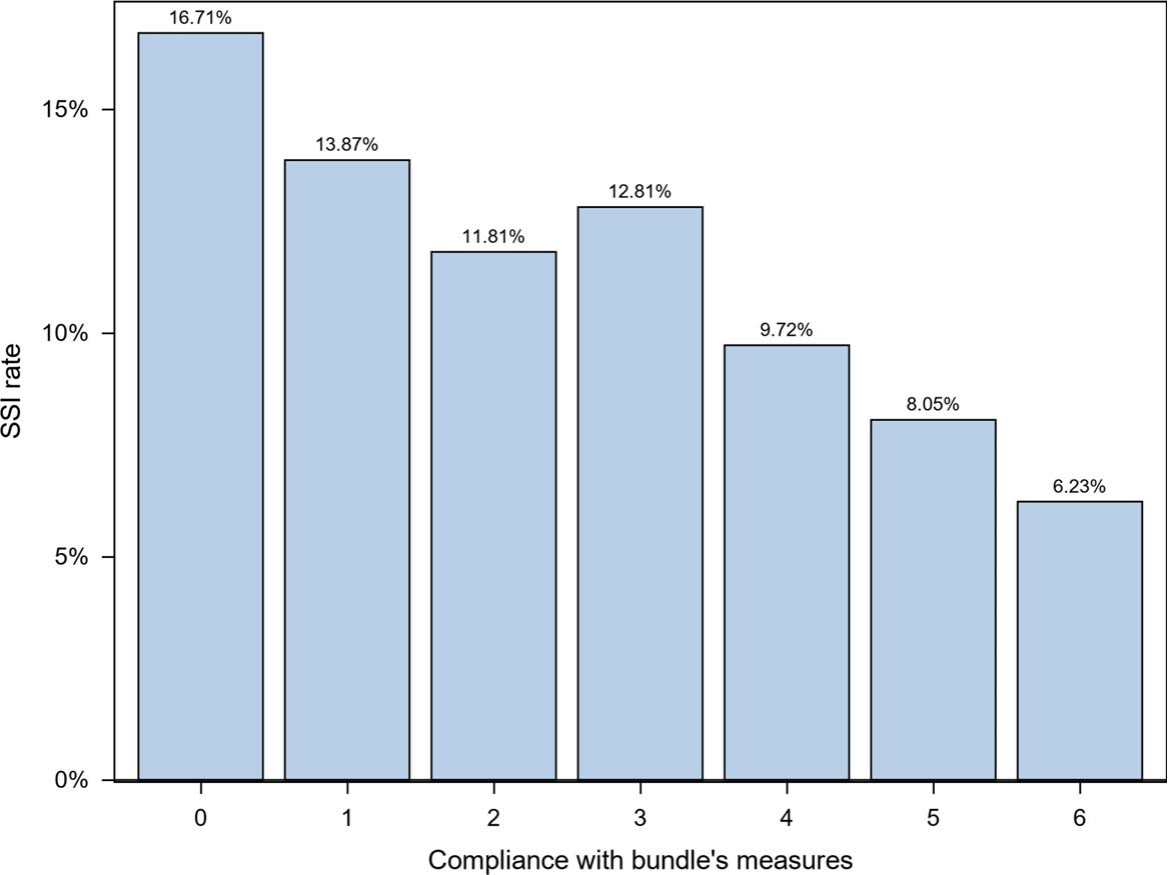
Colorectal SSI rates according to compliance with the elements included in the bundle. SSI, surgical site infection.

**Figure 4:**
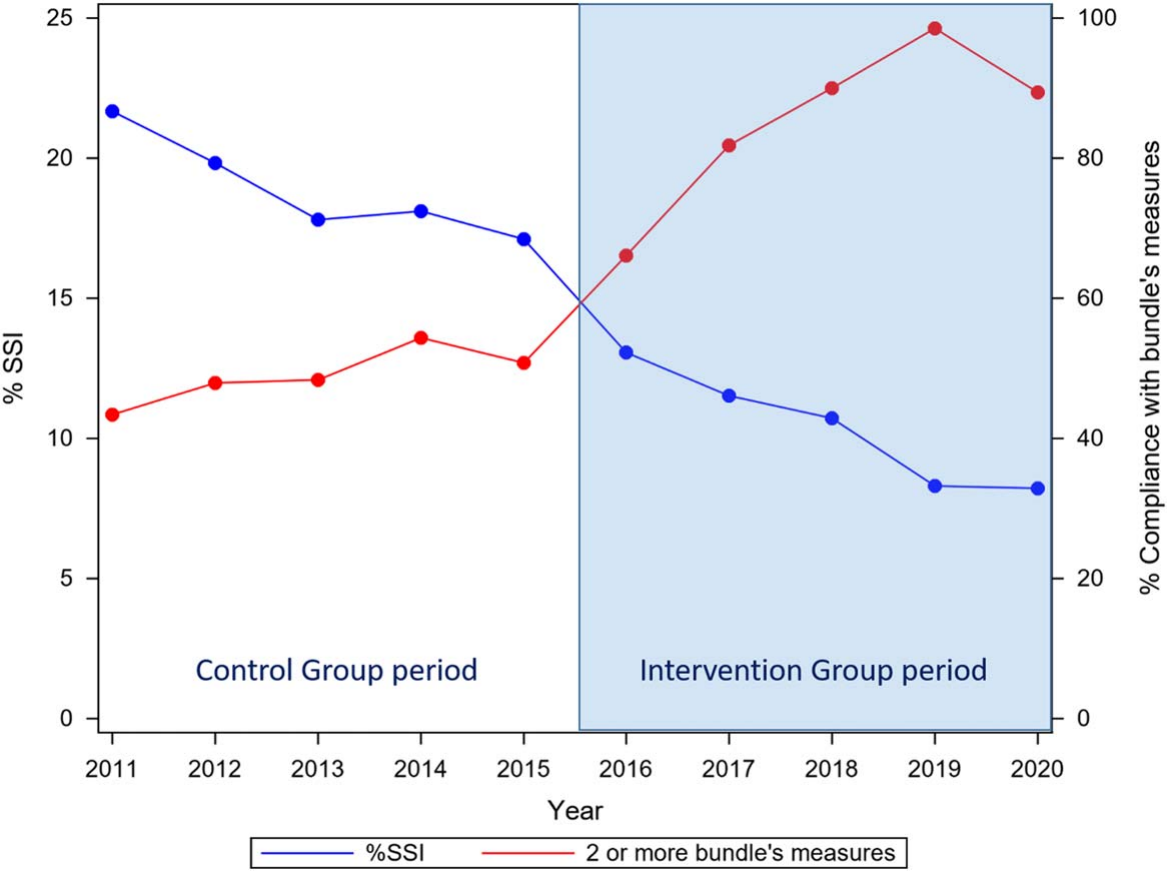
Relationship between the implementation of the elements of the bundle throughout the entire study period (covering the Control Group and the Intervention Group) and the evolution of the overall SSI rates. The most marked decrease in SSI occurred in 2016, the first year of the dissemination of the bundle. SSI, surgical site infection.

**Figure 5:**
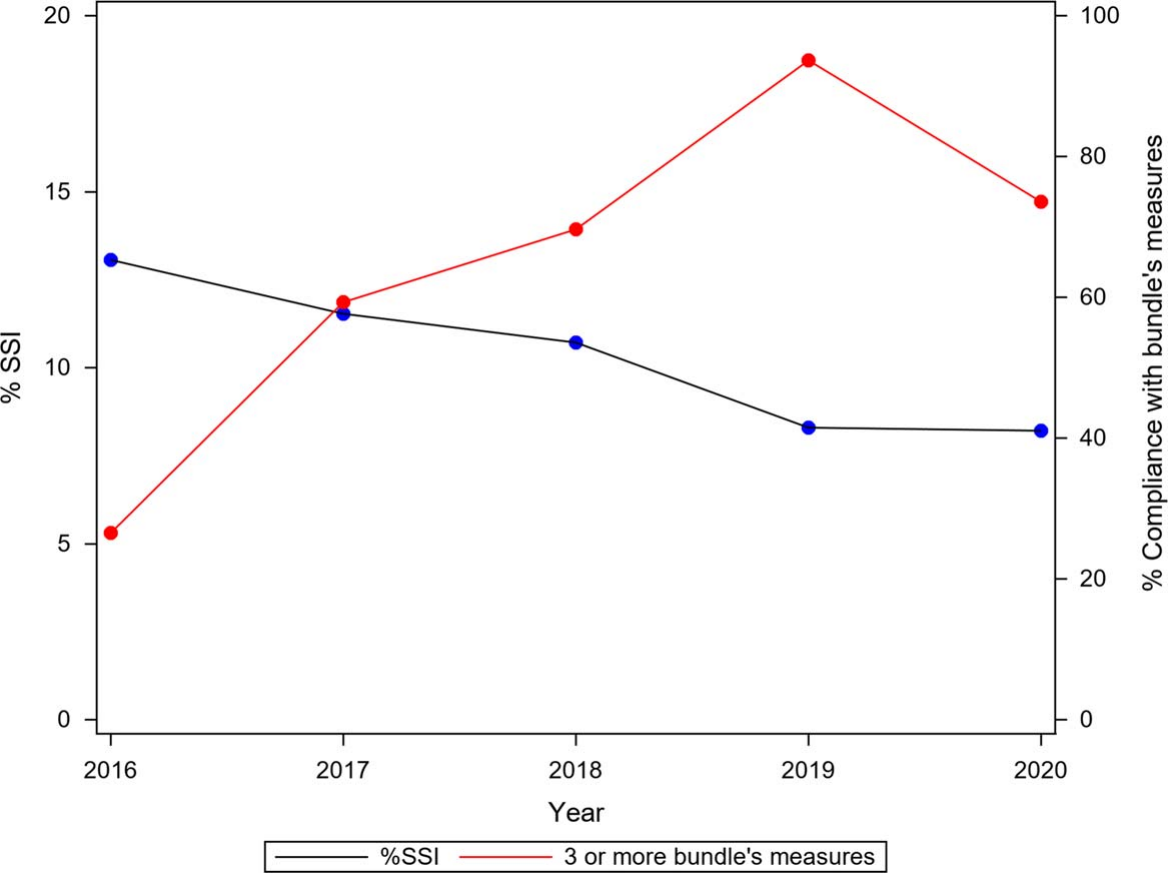
Relationship between compliance with the elements of the bundle and the evolution of overall SSI rates in the Intervention Group period (2016–2020). The information collected on compliance in 2020 may have been affected by the coronavirus disease 2019 pandemic. SSI, surgical site infection.

Overall SSI rates ranged from 16.71% when no bundle measures were used to 6.23% when all six measures were appropriately applied (Fig. 3). Bundle implementation reduced the probability of overall SSI (OR: 0.331; CI_95_: 0.242–0.453) and O/S-SSI (OR: 0.643; CI_95_: 0.416–0.919). Analyzing colon and rectal cases separately, the bundle effect was well maintained in colon surgery (overall SSI: OR, 0.273 [CI_95_: 0.188–0.395]; O/S-SSI, OR of 0.720 [CI_95_: 0.532–0.974]). In rectal surgery, however, its effect was less robust, OR of 0.545 [CI_95_: 0.301–0.985] for overall SSI and 0.626 [CI_95_: 0.425–0.923] for O/S-SSI. Figure 4 shows the relation between the increase in the implementation of the bundle elements over time and the decrease in overall SSI throughout the two periods of study. To show this relation more clearly, Figure 5 displays only the Intervention Group. In the first year of implementation of the bundle, a 19% drop in overall SSI rates was achieved, the largest annual fall recorded since surveillance began.

### Individual effect of bundle measures on SSI rates

**Figure 6:**
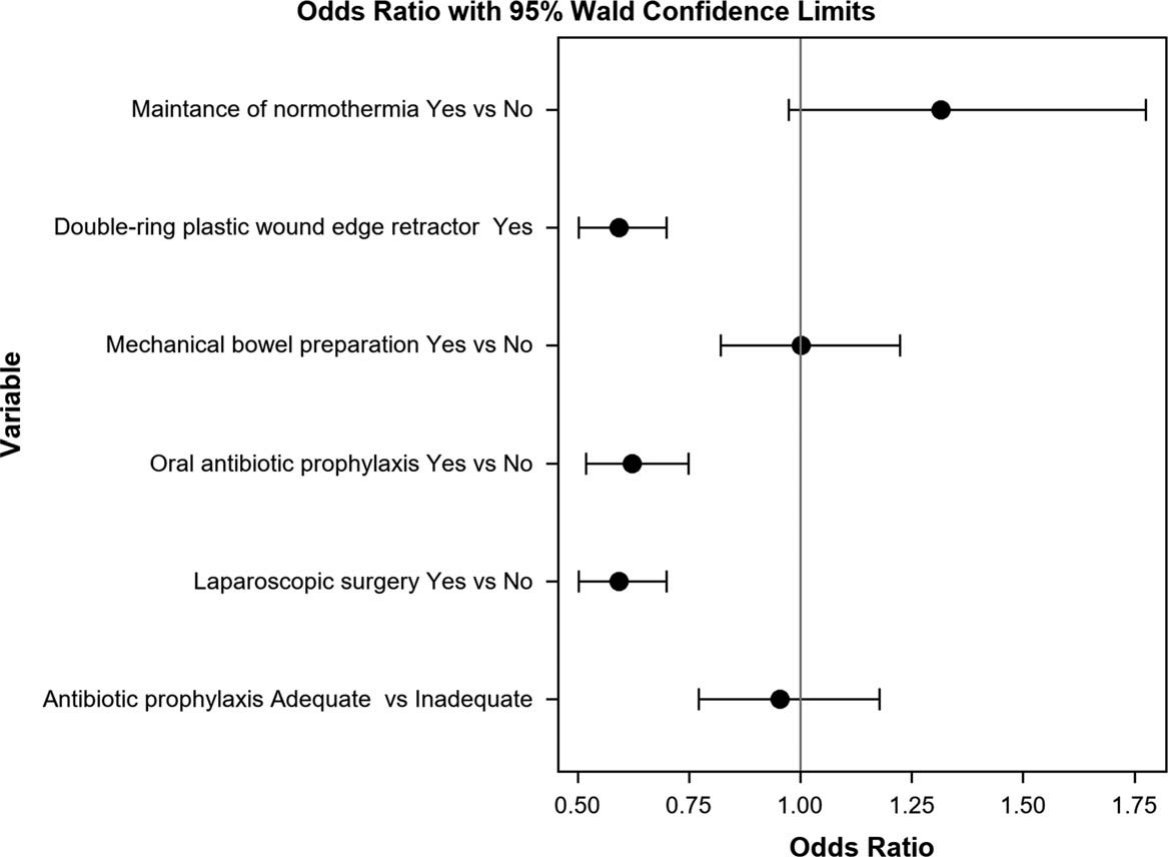
Multivariate analysis of the effect of the measures of the bundle on overall surgical site infection rates.

**Figure 7:**
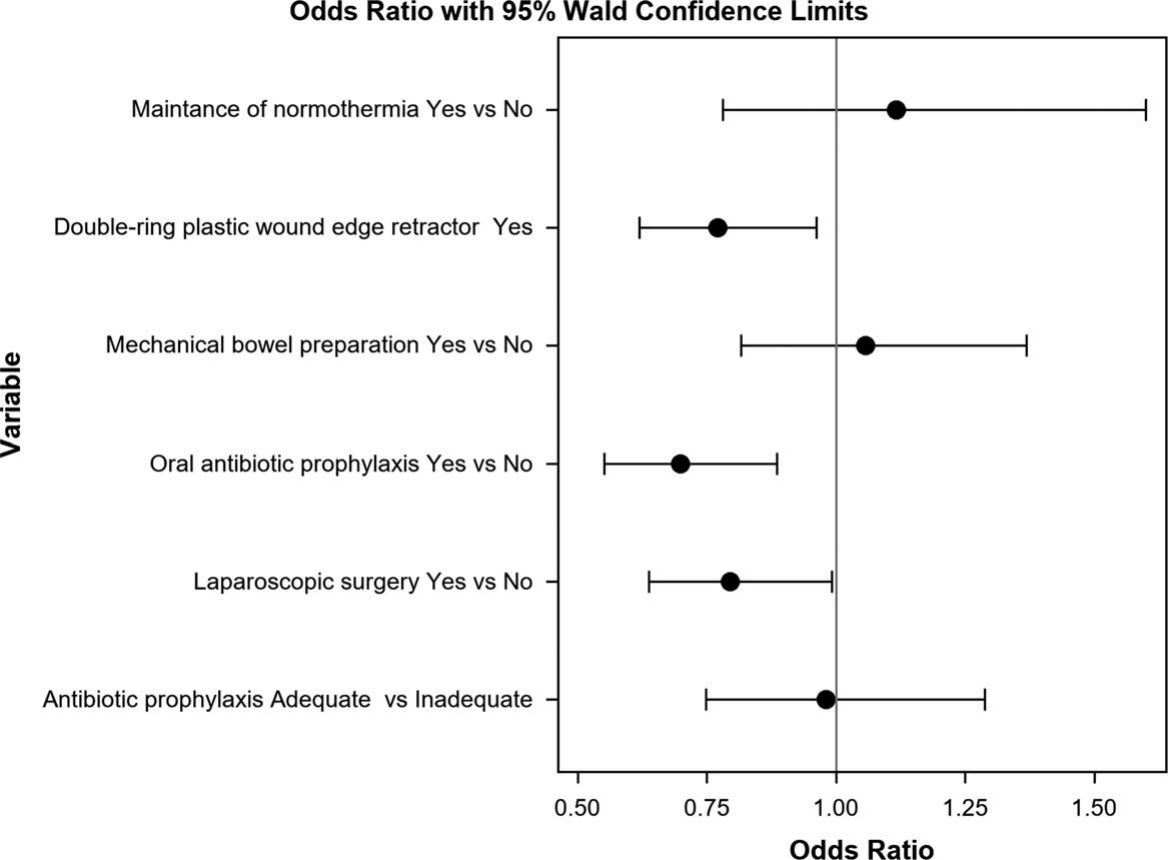
Multivariate analysis of the effect of the measures of the bundle on organ/space-surgical site infection rates.

In the univariate analysis of colon and rectal cases considered together (Table 6), all measures except for normothermia were associated with a decrease in overall SSI. For O/S-SSI, laparoscopy, OAP, and MBP were protective factors. Multivariate analysis confirmed that laparoscopy, OAP, and wound protectors decreased overall colorectal SSI (Fig. 6) and O/S-SSI (Fig. 7).

**Table 6:** Univariate and multivariate analysis of the effect of the bundle measures on SSI rates.

	Univariate			Multivariate		
	OR	CI_95_	*P*	OR	CI_95_	*P*
Colorectal overall SSI						
Adequate antibiotic prophylaxis	0.858	0.760–0.969	0.0139	0.953	0.771–1.180	0.6611
Laparoscopic surgery	0.561	0.507–0.621	<0.0001	0.592	0.501–0.700	<0.0001
Maintenance of normothermia			0.1597	1.315	0.973–1.777	0.0748
Oral antibiotic prophylaxis (OAP)	0.586	0.515–0.666	<0.0001	0.623	0.516–0.751	<0.0001
Mechanical bowel preparation (MBP)	0.720	0.627–0.827	<0.0001	1.002	0.819–1.225	0.9871
Double-ring wound retractor	0.660	0.576–0.755	<0.0001	0.592	0.500–0.701	<0.0001
Colorectal O/S-SSI						
Adequate antibiotic prophylaxis			0.6636	0.981	0.747–1.289	0.8903
Laparoscopic surgery	0.817	0.711–0.939	0.0045	0.795	0.637–0.993	0.0434
Maintenance of normothermia			0.6716	1.117	0.779–1.601	0.5482
OAP	0.664	0.563–0.784	<0.0001	0.699	0.551–0.888	0.0033
MBP	0.819	0.682–0.983	0.0322	1.057	0.816–1.369	0.6761
Double-ring wound retractor			0.1248	0.772	0.618–0.964	0.0224
Colon overall SSI						
Adequate antibiotic prophylaxis	0.804	0.692–0.933	0.0042	0.980	0.755–1.273	0.8822
Laparoscopic surgery	0.490	0.433–0.555	<0.0001	0.506	0.414–0.619	<0.0001
Maintenance of normothermia			0.0915	1.418	0.970–2.071	0.0713
OAP	0.473	0.405–0.553	<0.0001	0.577	0.463–0.718	<0.0001
MBP	0.582	0.497–0.683	<0.0001	0.896	0.714–1.124	0.3416
Double-ring wound retractor	0.733	0.614–0.876	<0.0006	0.683	0.548–0.851	<0.0007
Colon O/S-SSI						
Adequate antibiotic prophylaxis			0.9953	1.060	0.754–1.491	0.7381
Laparoscopic surgery	0.689	0.582–0.816	<0.0001	0.602	0.465–0.780	0.0001
Maintenance of normothermia			0.2504	1.345	0.836–2.164	0.2212
OAP	0.608	0.496–0.744	<0.0001	0.681	0.515–0.902	0.0072
MBP	0.697	0.565–0.859	0.0007	0.986	0.737–1.319	0.9238
Double-ring wound retractor			0.9257	0.937	0.696–1.261	0.6658
Rectal overall SSI						
Adequate antibiotic prophylaxis			0.9923	0.936	0.646–1.358	0.7291
Laparoscopic surgery	0.728	0.608–0.872	0.0006	0.804	0.585–1.104	0.1769
Maintenance of normothermia			0.8599	1.147	0.694–1.896	0.5936
OAP	0.736	0.576–0.939	0.0138	0.682	0.472–0.985	0.0415
MBP	0.666	0.467–0.950	0.0251	0.976	0.584–1.632	0.9263
Double-ring wound retractor	0.687	0.554–0.853	0.0007	0.598	0.453–0.789	0.0003
Rectal O/S-SSI						
Adequate antibiotic prophylaxis			0.6325	0.897	0.566–1.422	0.6443
Laparoscopic surgery			0.2933	1.585	0.992–2.532	0.0541
Maintenance of normothermia			0.4669	0.811	0.462–1.426	0.4676
OAP	0.651	0.482–0.880	0.0053	0.670	0.423–1.061	0.0875
MBP			0.1114	0.846	0.446–1.604	0.6088
Double-ring wound retractor			0.2866	0.733	0.514–1.045	0.0861

O/S-SSI, organ/space-surgical site infection; SSI, surgical site infection.

The results were similar when only colon surgery was analyzed. In the univariate analysis of rectal cases, MBP showed a protective effect on O/S-SSI but not on overall SSI. In the multivariate analysis, only systemic IV prophylaxis and plastic wound retractor were protective factors for overall SSI for rectal surgery, while none of the measures showed a significant effect on O/S-SSI (Table 6).

### Secondary variables

#### Median length of stay

The median postoperative LOS for the whole group was 7 days (IQR 5–11). A significant decrease was noted after the implementation of the bundle (*ρ*=−0.98414), with a fall from 8 days in the CG to 6 days in the IG (*P*<0.0001).

#### Time to SSI

No differences were detected between the groups in the median time elapsed between the intervention and the appearance of overall SSI, with medians of 7 days (IQR 5–12) vs. 8 days (IQR 5–13); *P*=0.2895. However, differences were found in O/S-SSI, with a median of 7 days (4–11) in the CG and 6 days (4–11.5) in the IG; *P*=0.0075.

#### Postdischarge SSI

Overall colorectal SSI was diagnosed during the first admission in 3856 (70.69%) cases and at postdischarge surveillance in 1596 patients (29.26%). In the latter group, 870 (54.5%) required readmission. Postdischarge SSI rates were 27.89% in the CG group and 32.09% in the IG group (*P*=0.0099). Readmission was also more frequent in the IG (15.21% vs. 17.40%; *P*=0.0099).

#### Mortality

Overall mortality was 1.12% and decreased significantly over the course of the study: from 1.49% in the CG to 0.80% in the IG for colorectal SSI (*P*<0.0001), from 1.67% to 0.86% for colon surgery (*P*<0.0001), and from 1.05% to 0.65% for rectal surgery (*P*=0.0203).

### Pathogens detected in SSI

An etiological diagnosis was achieved in 3840 patients with SSI (70.30%) (Table 7). There were 3620 microorganisms isolated from the 3612 SSI in the CG and 1525 from 1850 in the IG. Comparison of the two groups demonstrated differences only in the etiology of O/S-SSI, in which more Gram-positive cocci (22.07% vs. 36.41%), fewer Gram-negative bacteria (72.20% vs. 53.15%), and more fungi (2.38% vs. 6.41%) were isolated in the IG. In this group, the isolation of *Enterococcus faecalis*, *Enterococcus faecium*, and *Candida* spp. doubled in O/S-SSI.

**Table 7:** Comparison of etiology of incisional (I-SSI) and organ/space (O/S-SSI) colorectal surgical site infection according to the study group.

	Control group (%)	Intervention group (%)	*P*
Organisms in incisional SSI			
Gram-positive bacteria	31.76	35.38	0.0736
** ** *Enterococcus faecalis*	9.65	12.05	0.0678
** ** *Enterococcus faecium*	3.50	4.36	0.2972
** ** *Enterococcus* spp.	1.03	0.51	0.1935
** ** *MRSA*	0.86	1.67	0.00746
** **Others	16.71	16.79	0.9602
Gram-negative bacteria	61.92	59.62	0.2727
** ** *Escherichia coli*	36.24	29.87	0.0018
** ** *Klebsiella* spp.	3.91	4.87	0.2639
** ** *Pseudomonas* spp.	8.10	9.49	0.2486
** ** *Enterobacter* spp.	5.11	4.62	0.5954
** **Others	8.56	10.77	0.0764
Anaerobes	5.34	3.97	0.1422
** ** *Clostridium* spp.	0.17	0.51	0.1520
** ** *Bacteroides* spp.	5.17	3.46	0.0595
Yeasts	0.98	1.03	0.9083
** ** *Candida albicans*	0.98	1.03	0.9083
Organisms in organ/space-SSI			
Gram-positive bacteria	22.07	36.41	<0.0001
** ** *Enterococcus faecalis*	7.68	15.00	<0.0001
** ** *Enterococcus faecium*	7.02	13.59	<0.0001
** ** *Enterococcus* spp.	1.32	1.63	0.5064
** ** *MRSA*	0.40	0.54	0.5794
** **Others	5.65	5.65	0.9969
Gram-negative bacteria	72.20	53.15	<0.0001
** ** *Escherichia coli*	26.57	27.39	0.6339
** ** *Klebsiella* spp.	4.24	7.50	0.0002
** ** *Pseudomonas* spp.	5.83	6.63	0.3879
** ** *Enterobacter* spp.	3.40	4.46	0.1517
** **Others	32.17	7.17	<0.0001
Anaerobes	3.35	4.02	0.3557
** ** *Clostridium* spp.	0.62	0.65	0.9114
** ** *Bacteroides* spp.	2.74	3.37	0.3358
Yeasts	2.38	6.41	<0.0001
** ** *Candida albicans*	2.38	6.41	<0.0001

Incisional surgical site infection includes superficial-SSI and deep-SSI.

MRSA, methicillin-resistant *Staphylococcus aureus*.

## Discussion

This prospective cohort study provided strong support for the implementation of a colorectal SSI reduction bundle in a broad cohort of hospitals and demonstrated its efficacy in reducing SSI.

Little is known about the implementation of SSI preventive bundles in large groups of hospitals. Most of the colorectal bundles described to date have been implemented in single hospitals^35^; very few are regional or national bundles designed to be introduced at multiple centers. Previous research has highlighted that prevention bundles may be more difficult to introduce at multicenter level and that their clinical efficacy in this setting has not been demonstrated^27^.

Although several international guidelines regarding SSI prevention have been published^36^,^37^, guidelines are not self-implementing, and suboptimal compliance rates have been reported^38^,^39^, even in colorectal surgery^40^. Internal barriers to implementation are mainly related to human factors, while external barriers are environmental factors such as lack of leadership or organizational culture^41^,^42^. To overcome these difficulties, bundles of evidence-based interventions have been proposed^15^.

At least three meta-analyses have shown that when correct adherence to specific evidence-based bundles is achieved, SSI risk in colorectal surgery is reduced by an average of 40–50%^35^,^43^,^44^. However, these bundles are not homogeneous in terms of the measures included, and they are not widely used^21^. In some cases, even high compliance with the measures was not directly associated with reducing SSI rates^23^,^45^, and the adequate selection of the components of a given bundle is probably the key to its success^46^.

A systematic review^42^ studied the effect of implementation strategies on the prevention of SSI in abdominal surgery, defined as techniques designed to increase the adoption of health promotion activities^47^. The review showed that the highest risk reduction was achieved by applying a set of ‘top five’ activities: audit and feedback, organizational culture, monitoring the performance of healthcare delivery, reminders, and educational meetings. This bundle was successfully introduced in less than 1 year, leveraging a nationwide infection surveillance system that was already implementing these five strategies. The application of bundles in similar multicenter collaborative settings has shown that quality improvement studies should consider not only surgeon behavior, but also institutional traits for their optimal implementation^48^.

Most studies have analyzed colon and rectal surgeries together; separate assessments of patients undergoing colon and rectal surgery are scarce^49^,^50^. Although the risk factors and SSI rates of colon and rectal surgery differ^49^,^51^,^52^, it should be highlighted that the present bundle had an effect on both types of procedures.

More importantly, the bundle was effective at all three surgical sites, including the organ space site, where the consequences in terms of mortality and LOS are more severe than in I-SSI^53^,^54^. However, although most published colorectal bundles have demonstrated their beneficial effect on I-SSI, most of them did not improve rates of O/S-SSI^24^,^55^.

The observed reduction in SSI rates is likely due to the implementation of the bundle, in view of the strong association found between increasing bundle compliance and lower levels of SSI. The most efficient measures were OAP, laparoscopic surgery, and the use of a double-ring plastic wound retractor. The bundle’s efficacy in decreasing SSI rates was linearly correlated with the number of elements used. While some of the bundle measures appeared specifically designed to prevent either incisional or intra-abdominal infection, they worked together to reduce SSI at all levels. All of them showed individual efficacy for overall SSI prevention, except for maintenance of normothermia. For O/S-SSI, only laparoscopy, MBP, and OAP were effective. Multivariate analysis confirmed laparoscopy, OAP and wound retractor as protective factors against overall and O/S-SSI.

The relatively low impact of systemic antibiotic prophylaxis may be explained by the fact that only properly administered prophylaxis was considered for the analysis. The criteria used to consider it ‘adequate’ were very strict and comprised: the type of drug, dose, the timing of infusion, completion before surgical incision, and duration of therapy. Although prophylaxis was performed and recorded in all patients, a single deviation from the recommended guidelines was enough for the process to be considered inadequate.

The lack of effectiveness in maintaining body temperature may also seem surprising, but it should be noted that the difference in temperature between patients with and without infection was found to be 0.1°C. Seminal randomized clinical trials^56^,^57^ demonstrated the detrimental effect of severe hypothermia (around 34°C) on SSI rate after colorectal surgery and led to the current recommendation of keeping a core body temperature above 36°C in the perioperative period. However, subsequent cohort studies and a meta-analysis^58^ found no association between perioperative hypothermia and SSI risk. It should be noted that the differences between normothermic and hypothermic patients in the original studies^56^,^57^ were in the order of 1–2°C. In contrast, the differences in the cohort studies that reported negative results had an average of 0.1°C, as observed in this study. Since today the vast majority of patients are actively warmed, it is likely that these minor temperature differences between those with SSI and those without will no longer be statistically significant.

OAP and MBP are controversial SSI preventive measures that are exclusively used for colorectal surgery^59^–^61^. Although there is broad consensus that intravenous antibiotic prophylaxis is essential before colorectal surgery, it is still debated whether oral antibiotics should be added. In addition, the development of multimodal rehabilitation programs^62^ and the publication of several conflicting studies has fueled the controversy surrounding MBP and its potential combination with OAP, leading to a significant decrease in their prescription rates worldwide. In 2017, a European survey recorded an oral prophylaxis use of only 11% and routine use of MBP of 29.6%^63^.

When designing the bundle, a multidisciplinary team decided to include OAP combined with MBP (mechanical and oral antibiotic bowel preparation, MOABP). Subsequently, two randomized trials compared MOABP^64^ or OAP^65^ with no bowel preparation, the first of which found no differences in SSI rate and the second only reduction in S-SSI rates. While waiting for the confirmation of these results, some researchers think that the MOABP strategy should be continued, albeit with the adjustments made necessary by the new findings in the gut microbiome^66^. Recent guidelines of several scientific societies have recommended the inclusion of OAP in their bundles for colorectal surgery, even in the setting of Enhanced Recovery After Surgery programs^67^,^68^.

After the implementation of the bundle, increases in *E. faecalis*, *E. faecium*, *Klebsiella* spp., and *Candida albicans* were detected in O/S-SSI. It could be argued that this change in the infecting flora is due to the administration of OAP. In experiments with mice, oral administration of antibiotics, including neomycin, changed the composition of the gut microbiota and increased the abundance of potentially pathogenic genera such as *Enterococcus* 69. Other authors have documented a risk of selection of resistant *Enterobacteriaceae* after treatment with oral colistin and neomycin^70^. Similarly, another study found that intestinal preparation with erythromycin and neomycin may be an independent risk factor for the selection of nosocomial strains of enterococci^71^.

### Strengths and limitations of the study

This study has several limitations. First, even though the sequential groups are, to some extent, homogeneous, certain changes in practices during the time frame of the study, such as the increased use of laparoscopy, may have interfered with the results. However, the pragmatic nature of the study and the fact that it was carried out within a consolidated infection surveillance program allowed prospective recording of the data from the two study groups and the use of the same methodology. Second, the improvement in the results may be due only to the surveillance program itself. Surveillance activities are known to reduce the tendency of healthcare-associated infections^72^, although the surveillance effect lasts only a few years^73^, and in most cases, it is difficult to disentangle it from the result of implementing specific interventions. In this case, the decline in SSI rates appears to be related to the introduction of the bundle, as reported elsewhere^74^. Third, as in similar nationwide databases, the number of variables collected was restricted, and some risk factors, such as BMI, smoking, and diabetes, or secondary outcomes, such as anastomotic leakage, were not evaluated. Finally, the level of compliance with some of the bundle measures was uneven at the participating hospitals. The strengths of the study include its large number of cases followed up prospectively as part of a consolidated program, which means that its results can probably be extrapolated to other settings.

### Implications

The current study describes the successful prospective implementation of a comprehensive SSI prevention bundle in a large, diverse network of hospitals. The opportunity to leverage a bundle of this kind within a long-established surveillance program allowed its controlled implementation in a short period of time and the use of a large prospective database to analyze the clinical outcomes. The study provides a pragmatic insight into bundle implementation as well as clinical evidence to further the efforts to reduce SSI.

## Conclusions

These results show that a common series of measures can be successfully introduced in the setting of a nationwide healthcare-related infection surveillance system. The proposed bundle, including OAP, decreased overall SSI, O/S-SSI, LOS, and mortality, both in the elective colon and rectal surgery in a wide population of patients undergoing elective procedures. The implementation of the bundle halved the OR for SSI. Preoperative OAP, the use of a double-ring plastic wound retractor, and the laparoscopic technique were the measures with the strongest impact on outcomes.

## Ethical approval

Data extraction was approved by the Institutional Research Board with code 20166009, and the study was approved by the Clinical Research Ethics Committee of Hospital General de Granollers with code 2021006. The need for informed consent and the provision of an information sheet were waived because data were routinely collected as part of hospitals surveillance and quality improvement.

## Sources of funding

This study received no external funding. The VINCat Surveillance Program, from which the data was obtained, is supported by public funding from the Catalan Health Service, Department of Health, Generalitat de Catalunya.

## Author contribution

F.G., M.P., J.M.B., N.A.: conceptualization and design; A.V., A.A., and J.M.B.: methodology; E.L., A.V., M.P.: acquisition of data; A.V., A.A., E.L., J.M.B., N.A., and M.P.: formal analysis and interpretation of data; J.M.B. and N.A.: drafting of the manuscript – original draft preparation; J.M.B., M.P., N.A., A.A., D.P., M.P., A.V., M.P.-A., D.F., D.P., A.A.-T., M.P., M.P.-A., A.S.-P., E.L., and F.G.: critical revision of the manuscript.

## Conflicts of interest disclosure

The authors declare no conflict of interest. All authors submitted the ICMJE Form for Disclosure of Potential Conflicts of Interest.

## Research registration unique identifying number (UIN)

1. Name of the registry: Research Registry.
2. Unique identifying number or registration ID: researchregistry8407.
3. Hyperlink: www.researchregistry.com, https://www.researchregistry.com/browse-the-registry#home/registrationdetails/634d398305178e002191c978/
4. Name of the registry: ClinicalTrials.gov
5. Identifier: NCT04129177
6. Hyperlink: https://clinicaltrials.gov/ct2/show/NCT04129177.

## Guarantor

Guarantor: J.M. Badia.

## Data availability statement

The research data is prospectively registered and belongs to the Nosocomial Infection Surveillance System in Catalonia (VINCat), a program from the Catalan Health Service, Department of Health, Generalitat de Catalunya. Anonymous data extraction was approved by the Institutional Research Board of the VINCat. All data will be made available on request.

## Provenance and peer review

Not commissioned, externally peer-reviewed.

## Appendix 1. Members of Infection Control Teams participating in the program

Dolors Castellana and Elisa Montiu González, Hospital Universitari Arnau de Vilanova de Lleida; Graciano García Pardo and Francesc Feliu Villaró, Hospital Universitari Joan XXIII de Tarragona; Josep Rebull Fatsini and M. France Domènech Spaneda, Hospital Verge de la Cinta de Tortosa; Marta Conde Galí and Anna Oller Pérez-Hita, Hospital Universitari Dr. Josep Trueta Girona; Lydia Martín and Ana Lerida, Hospital de Viladecans; Sebastiano Biondo and Emilio Jiménez Martínez, Hospital Universitari de Bellvitge; Nieves Sopena Galindo and Ignasi Camps Ausàs, Hospital Universitari Germans Tries i Pujol; Carmen Ferrer and Luis Salas, Hospital Universitari Vall d’Hebron; Rafael Pérez Vidal and Dolors Mas Rubio, Althaia Xarxa Assistencial de Manresa; Irene García de la Red, Hospital HM Delfos; Mª Angels Iruela Castillo and Eva Palau i Gil, Clínica Girona; José Antonio Martínez Martínez and Mª Blanca Torralbo Navarro, Hospital Clínic de Barcelona; Maria López and Carol Porta, Hospital Universitari Mútua de Terrassa; Alex Smithson Amat and Guillen Vidal Escudero, Fundació Hospital de l'Esperit Sant; José Carlos de la Fuente Redondo and Montse Rovira Espés, Hospital Comarcal Mora d'Ebre; Arantxa Mera Fidalgo and Luis Escudero Almazán, Hospital de Palamós; Monserrat Ortega Raya and Aina Gomila, Hospital Parc Taulí de Sabadell; Vicens Diaz-Brito and Mª Carmen Álvarez Moya, Parc Sanitari Sant Joan de Déu (Hospital de Sant Boi); Laura Grau Palafox and Yésika Angulo Gómez, Hospital de Terrassa; Anna Besolí Codina and Carme Autet Ricard, Consorci Hospitalari de Vic; Carlota Hidalgo López and Marta Pascual Damieta, Hospital del Mar; Jordi Cuquet Pedragosa and Demelsa Mª Maldonado López, Hospital General de Granollers; David Blancas and Esther Moreno Rubio, Consorci Sanitari del Garraf; Roser Ferrer i Aguilera, Hospital Sant Jaume de Calella; Simona Iftimie Iftimie and Antoni Castro-Salomó, Hospital Universitari Sant Joan de Reus; Rosa Laplace Enguídanos and Maria Carmen Sabidó Serra, Hospital de Sant Pau i Santa Tecla; Núria Bosch Ros, Hospital de Santa Caterina; Virginia Pomar Solchaga and Marta Piriz Marabaján, Hospital de la Santa Creu i Sant Pau; Laura Lázaro Garcia and Angeles Boleko Ribas, Hospital Universitari Quirón Dexeus; Jordi Palacín Luque and Alexandra Lucía Moise, Pius Hospital de Valls; Mª Carmen Fernández Palomares and Santiago Barba Sopeña, Hospital Universitari Sagrat Cor; Eduardo Sáez Huertas and Sara Burges Estada, Clínica NovAliança; Josep María Tricas Leris and Eva Redon Ruiz, Fundació privada Hospital de Mollet; Montse Brugués Brugués and Susana Otero Acedo, Consorci Sanitari de l’Anoia. Igualada; Maria Cuscó Esteve and Lourdes Gabarró, Hospital Comarcal de l’Alt Penedès; Fco. José Vargas-Machuca and Mª de Gracia García Ramírez, Centre MQ de Reus; Elena Vidal Díez and Ana Maria Ciscar Bellés, Consorci Hospitalari del Maresme. Hospital de Mataró; Mariló Marimón Morón and Marisol Martínez Sáez, Hospital Universitari General de Catalunya; Josep Farguell and Mireia Saballs, QUIRON Salud; Montserrat Vaqué Franco and Leonor Invernón Garcia, Hospital de Barcelona; Rosa Laplace Enguídanos and Meritxell Guillemat Marrugat, Hospital Comarcal del Vendrell; Ana Coloma Conde and Lucrecia López González, Hospital Moisès Broggi.
